# Investigating discrepancies in perceptions regarding the provision of hospital rest and relaxation spaces in Scotland during the COVID-19 pandemic and beyond: a qualitative study

**DOI:** 10.1136/bmjopen-2024-096154

**Published:** 2025-08-12

**Authors:** Kim A Walker, Kathryn B Cunningham, Julie Ferguson, Kathrine Gibson Smith, Gillian Marion Scanlan, Joanne E Cecil, Lisi Gordon, Anita Laidlaw, Lindsey Margaret Pope, Peter Johnston, Gill Aitken

**Affiliations:** 1 CHERI, University of Aberdeen, Aberdeen, UK; 2 University of Dundee, Dundee, UK; 3 NHS Education for Scotland, Edinburgh, UK; 4 University of St Andrews, St Andrews, UK; 5 Heriott-Watt University, Edinburgh, UK; 6 University of Glasgow, Glasgow, UK; 7 The University of Edinburgh, Edinburgh, UK

**Keywords:** Organisation of health services, COVID-19, Health Workforce

## Abstract

**Abstract:**

**Objectives:**

To investigate discrepancies in perceptions regarding the accessibility and availability of rest and relaxation (R&R) spaces between hospital doctors in Scotland and NHS Scotland regional health boards (HBs), with the intention of informing best practices for organisational policy on the provision of R&R spaces both now and in the future.

**Design:**

A qualitative study, through an inhabited institutionalism (II) lens, of semi-structured interviews of hospital doctors across the career continuum in Scotland and all NHS regional HBs in Scotland providing written information relating to R&R space provision.

**Setting:**

NHS Scotland during the COVID-19 pandemic and beyond.

**Participants:**

Hospital doctors (n=30) who had participated in a larger qualitative study and provided specific insights on R&R spaces. All NHS Scotland regional HBs (n=14).

**Results:**

Although HBs reported the provision of R&R spaces, numerous doctors reported R&R spaces had been removed, relocated or were inaccessible. Furthermore, limited awareness of their availability attributed to inadequate communication, compounded the issue. This divergence between institutional reporting and front-line experience can be interpreted through the lens of II, which posits that institutional polices are often interpreted and implemented differently.

**Conclusions:**

This study emphasises how crucial R&R spaces are to promoting doctors’ well-being especially during the time of high stress. HBs must not only guarantee the accessibility and physical availability of R&R spaces but also enhance their communication regarding the provision.

**Strengths and limitations of this study**

- This was a theory-based national study in Scotland, relevant for the well-being, productivity and workforce retention of hospital doctors across the career continuum.
- The study was a large qualitative study using mixed methods, delivering impactful results relevant to the current pressures of the health service.
- All interviews with the hospital doctors were completed before all the information was received from Health Boards (HBs), whereas the HBs were surveyed after the pandemic but were asked about past, present and future provision.
- Recruitment to the study of the hospital doctors was through a voluntary sign-up, which may bias opinion towards those who have issues but have no avenue for reporting them.

## Introduction

 There is much comment and debate about doctors’ well-being and how it can be supported, especially physical and psychological wellbeing.^1 2^ However, the literature prior to the pandemic lacked an evidence base for design and implementation of wellbeing interventions in difficult circumstances.^3^ The Scottish Doctors Wellbeing Study (SDWS),^4^ undertaken in Scotland in 2020–21, has developed evidence-based and theory-informed interventions to support doctors’ wellbeing during and beyond the pandemic. In this broader work, participants highlighted the role of rest and relaxation (R&R) spaces in supporting their physical, psychological, social and cultural wellbeing.^5^ However, an apparent discrepancy was noted between the perceptions of doctors and their employing organisation on the provision of these, and this paper aims to explore this disconnect further.

Provision of R&R spaces for staff to access has been identified as a key basic need for workers’ wellbeing^6^,^8^ and is recognised in the British Medical Association Charter.^9^ Throughout this paper, we use the term R&R space to represent a physical space provided free of charge by the employing institutions offering a physically and emotionally safe environment for hospital staff to freely express themselves without fear of prejudice or negative judgement. In essence, a shared social place or safe physical space away from patients, their relatives and public.^10^ Other terms have been used during and after COVID-19, for example, wellbeing hub, wobble rooms and rest rooms.^11^,^13^ The provision of these spaces and their accessibility is emphasised in healthcare workforce planning documents. The NHS Recovery Plan for the NHS Scotland workforce (2021, p7)^14^ states:

We will offer further practical support for the physical and emotional needs of the workforce—this will include additional funding for rest area, guidance to promote effective wellbeing conversation, new opportunities for staff to reflect on the emotional aspects of their work and further resources so staff at all levels can access peer support.

This inclusion reflects the increasing recognition and evidence that poor wellbeing in doctors presents challenges to patient safety, patient satisfaction, professionalism and workforce retention.^715^,^17^ Organisational messaging around care of staff wellbeing is often centred on individual physical and psychological wellbeing, placing responsibility for maintenance of wellbeing on individuals.^18^ However, it is now recognised that organisations are also central to supporting doctors’ wellbeing rather than relying solely on the individual.^5 9 19^

The health service structure in Scotland includes 14 regional health boards (HBs) with responsibility for employing doctors and provision for primary, secondary and tertiary care.

In the past, R&R physical spaces were the norm, but over the past two decades prior to the pandemic, these reduced as the demand for clinical space increased.^20^ The pandemic saw R&R spaces being (re)introduced by organisations as the importance of a supportive workplace environment was recognised alongside the negative impact of poor organisational culture on doctors’ wellbeing.^4 5 10^

The aim of the study in this paper was to investigate the discrepancies in perception of hospital doctors and their employing organisations, the NHS regional HBs, regarding provision of R&R spaces during the COVID-19 pandemic and beyond.

## Methods

### Context

The SDWS was a multi-phase interview study (an initial interview with two follow-up interviews) of the experiences of 100 doctors, across all career grades during a year of the pandemic.^5^ A preliminary analysis of the first interviews highlighted the value of R&R physical spaces away from the clinical environment. However, at follow-up interviews, many participants perceived their R&R spaces had been removed as time progressed. Therefore, as part of the subsequent interventional phase of the research project, we considered this issue in more detail, which is reported in this manuscript.

### Participant data collection

#### Hospital doctors

53% (n=53) of the doctors from the initial interviews (n=100) in the SDWS made specific reference to R&R spaces. Interviewees’ reference was the inclusion criteria. Specific data collection was undertaken as part of the second and third interviews from doctors who provided detailed information about R&R spaces. The key question in relation to this study was: *Do you currently have access to an R&R space?* The qualitative nature of the interviews allowed participants to expand on their answers, including consideration of the availability, and perhaps more crucially, the accessibility of these spaces in their workplace. 30 doctors provided specific responses to this question, and their data were included in the final analysis.

#### NHS Scotland regional HBs

Following on from the first interview data analysis, an infographic was developed highlighting the perceived benefits of R&R spaces for hospital doctors. This infographic, accompanied by a persuasion document, was sent via email to each HB; the Chief Executive, copied to the Medical Director and Director of Medical Education. This email outlined the importance and value placed on these safe spaces by the doctors and asked for their reinstatement where they had been removed (see online supplemental information associated with this paper).

Initial responses received to this request from some of the HB’s indicated they considered the spaces were still available, although in some circumstances, the physical location had changed. On further examination of the data, the existence of discrepancies between what the HBs were reporting was available and the perception of the doctors in those HBs was established.

Following the final interviews with the doctors, all HBs were emailed again asking about provision of the R&R spaces (box 1). A follow-up email was sent a month later, and any HB who had not responded to the initial or follow-up contact was then sent a Freedom of Information request for the same data to which they responded with the relevant information.

Box 1 **Request for information on rest and relaxation (R&R) spaces**

**What R&R spaces are currently available in your NHS Board?**

- Please include details of where each space is located within the hospital, the specific provision available and the opening hours.

**Are these R&R spaces intended to be permanent or temporary?**

- If temporary, please provide details of which are to be removed and when

**Have any R&R spaces been withdrawn during the pandemic?**

**Have any R&R spaces moved location during the pandemic?**

**Who can use these R&R spaces?**

- eg, staff members (all or particular groups), patients, patient families

**How are staff alerted to the presence of R&R spaces?**

### Data analysis

Initial framework analysis^21^ was used across the larger data set of doctors’ transcripts in NVivo to identify broad themes. This was undertaken by the research team members familiar with the data (KAW, LG, GS and JF) and was considered further through regular team discussion (KGS, KBC, AL, JC, LP, PJ and GA).

Qualitative interview transcripts relating to R&R spaces (n=30 doctors) and open-ended questionnaire/Freedom of Information request data (n=14 HBs) were analysed using content analysis.^22^ ^23^ The coding framework was constructed both deductively from the questions asked (eg, access to R&R space) and inductively from the data itself (eg, lack of communication regarding R&R space) in order that both the questions asked and the data itself drove the analysis. Analysis was undertaken by KBC with JF checking the interpretation of the data.

Inhabited Institutionalism (II) was adopted as the theoretical lens through which to further explore the data. Primarily, this choice allowed us to look at both the enactment of organisational change and how this was experienced by those influenced by the initiatives. The theoretical lens of II is doubly constructed^24^ in that it enables consideration of both the bigger picture and more local settings; looking both at how change is managed in the ways in which institutions provide guidelines and resources but also at how meaning is constructed and change enacted by ‘staff on the ground’. Institutions enact legitimate power, often with pressures towards homogeneity and conformity in the world where the strategies of New Public Management^25^ dominate, highlighting concerns such as a customer focus, audit and target setting; efficiency measures and performance reporting.^26^ Thus, II is a useful lens through which to consider healthcare management and the impact of decision-making on employees. People enact change, not organisations. II allowed us to consider not just how HBs reacted through their provision and maintenance of R&R spaces but also how these spaces were perceived and used by doctors.

II moves away from traditional views of organisation sociology where individuals are seen as nested within formal structures within organisations and seeks out the spaces that people inhabit in organisations, where change can be negotiated and enacted. The value of R&R spaces to those undertaking stressful and busy work, such as doctors, is easily predicted and understood. Using II as a theoretical lens widens our gaze to consider how these spaces might also function as relational spaces where conversation, socialisation and negotiations occur that may have upward (and outward) influence on institution logics and culture.^27^ Thus, by helping to reveal the links and interactions (termed coupling in II methodology) between structures and the inhabited spaces individuals occupy within institutions where changes can occur, II allows us to investigate how dynamic change happens (or is hindered) at senior and middle management level within institutions.^24^ For example, government bodies and HBs may have decided that R&R spaces should be provided, but the reality of where these spaces are situated and what they provide is decided at local level.^10^ Thus, the action of those within organisations will influence how tightly an organisation’s response to R&R spaces is aligned (coupled) to policy decisions and will be determined (to an extent) by the logics (or conventions, ie, the dominant narrative within the organisation)^28^ that are at play within individual institutions. The lens of II offers valuable insight into this area, where the topic of interest covers the enactment of policy and how this is perceived by those affected in the workplace. In considering how policy is interpreted and enacted, we also see how it is subverted in the clinical workplace.

The two datasets were then systematically compared for each regional HB, with specific reference to coupling and to logic as described in II by GA and checked by JF.

## Results

### Findings of analysis

#### Hospital doctors

30 doctors, all of whom had commented on R&R spaces in their first interview, provided specific information relating to R&R spaces in their workspace at their follow-up interviews. The key outcomes from the analysis were as follows: they have no provision and/or reported barriers to access especially the ability to reach them from some parts of the hospital during breaks; the poor communication relating to the location of the R&R spaces; and the lack of standard terminology and criteria for an R&R space. These doctors worked in 11 of the HBs.

#### HBs

100% of the HBs reported provision of R&R spaces for all staff across all sites (table 1). It was apparent from the responses that, again, the lack of standard terminology, what constitutes a R&R space and what is provided was very varied. The majority stated that where it was temporary, they hoped to secure funding to make it permanent, with only three HBs stating spaces had been removed.

**Table 1:** Regional health board participants: data of rest and relaxation provision

NHS board	Are R&R spaces available	Are they temporary or permanent	Have R&R spaces been withdrawn	Have R&R spaces been moved	Who can use these R&R spaces	How are staff alerted to R&R spaces
A	Yes	Temporary	No	New permanent spaces being constructed	Staff	Staff communications
B	Yes	Mostly permanent	No	No	Staff	Staff communications
C	Yes	Information not held	Information not held	No	Staff-only areas	Line manager communication
D	Yes	Temporary but intended to be permanent through endowment funding	No	Some	All staff for most of the areas	Staff communications and line manager
E	Yes—primarily cafes	Temporary and permanent	No	No	Some staff only, some for all	Staff communications
F	Yes	Intended to be permanent	No	No	Staff	Staff communications
G	Yes	Currently temporary, should be permanent if still required	No	Yes	Staff	Staff communications
H	Yes	Permanent	In one site	No	Staff	Staff communications
I	Yes	Temporary at present	Yes	Yes	Staff	Staff communications
J	Yes	Temporary and permanent	Yes	No	Staff	Staff communications
K	Yes	Intention to keep with charitable funding	No	Yes	Staff	Staff communications —newsletters
L	Yes	Some temporary	Unknown	Unknown	Some staff only	Unknown
M	Yes	Temporary but to be reviewed	No	No	All staff but now patient as well	Staff communications
N	Yes	Temporary	No	No	Majority – staff	Staff communications

R&R, rest and relaxation.

There was a high degree of unanimity between the HBs when discussing the provision of R&R spaces, but much more variation was reported in the hospital doctors’ experiences of such spaces. The majority of R&R spaces were reported by HBs as open to all staff. Only two mentioned a doctors’ mess space specifically for use by trainees which often attracts a fee to access/use them.

The main route for informing staff, including doctors, about the provision of R&R spaces was through staff communication including via email, online well-being pages, posters, dissemination through existing staff networks and meetings, inductions, word of mouth, newsletters and staff forums. Two HBs reported communications through the line manager route.

### Findings within II

#### Coupling

The provision of R&R spaces in hospitals has been shown to be loosely coupled with the policy and plans of the NHS. Hospital estates were acknowledged to be under intense pressure and often limited the space available for staff:

…we are actively looking for more indoor space to re-purpose for staff areas, but like all sites we have extremely limited space available that is not already being used. (HB)

Doctors’ experiences were at odds with the claims of all HBs that R&R spaces are available. Some doctors reported that they had no access to such spaces or that such spaces had been closed due to health and safety concerns.

It seems a lot of those initiatives that were really helpful for wellbeing and things, just because coronavirus has gone, doesn’t mean to say that the work we do is any less difficult or horrible and all those things for wellbeing, you think, why can’t we continue them? The fact they have already been stopped, there’s not really a willingness amongst management to continue them….Its those little basic things for people make a big difference. (Hospital doctor)

As stated earlier, the provision and what is provided in a R&R space can be variable. There were various terms used for these facilities, what was expected and what was provided was different. The HBs considered that all dining areas/cafes were R&R spaces even if being used by patients and the public. However, doctors mostly considered an R&R space to be a ‘backstage location’^5^ somewhere away from patients. In some cases, it was not clear if the R&R spaces were introduced because of the pandemic need or had been in place prior to the pandemic, for example, doctors’ mess. Provision altered over time across the HB area and even in individual hospitals.

….some individual teams and departments have their own kitchen areas, tea rooms etc. This provision is ad-hoc and not equitable across the site or staff groups. (HBd)

Doctors articulated that many of the issues around provision already existed, with the pandemic amplifying existing frustrations and lack of access to these R&R spaces impacting on their sense of belonging within the workplace.

there’s one staffroom basically in (hospital) and it is in the management corridor, so it is right next door to the clinical area manager and all that kind of thing and their secretaires and they use it all the time for going in to boil the kettle and stick things in the microwave and the staff hardly ever use it. It’s not a comfortable place. It’s pretty cold. It’s not very welcoming. It’s got round café-type tables with very shaky chairs, its got two couches that the managers tend to go and have their lunch sitting there. So why would staff go there? And that is the only staff facilities that there are. (Hospital doctor)

Even when space was made available, a barrier to staff using these spaces was associated with the perceived inaccessibility of some locations (ie, too far away or across the road from the main hospital) in which they are placed and/or poorly publicised to staff. In the five cases in which doctors’ noted non-availability, the four HBs involved reported provision of multiple spaces.

One thing about our one is, it’s not in the main hospital; you have to walk across to it and I don’t think it’s been advertised particularly well because quite a lot of people who I’ve spoken to about it, don’t know about it. Like, I only heard of it through word of mouth. (Hospital doctor)

Pressures on accommodation and financial concerns meant spaces such as cafes were often shared with patients and hospital visitors. This was clearly at odds with staff’s stated need for an area where they could decompress away from patients.

We have nowhere, or had nowhere, apart from the junior doctors’ mess. We had no designated outdoor space wither. As a result of COVID, and donations, there have been some benches which have been placed in an outdoor are, which has been made available for staff only which has been greatly used which I think just tells you there was a need there. I’ve said for a very long time that I think there should be an area of the dining room which should be staff only, but that’s been rejected time and time again as not being egalitarian and we can’t do that and also the dining room makes money from the public. (Hospital doctor)

#### Logic

Prior to the pandemic, institutional logics suggested that it was widely perceived spaces were not seen as important to those planning hospital estates, with most doctors interviewed noting that dedicated space for staff was uncommon, particularly in newer hospitals where staff noted there were often no dedicated spaces away from patients. Whether intended, or not, the message staff took from this was they were not valued in their workplace.

If I think about the two buildings that I work in, I think the staff are thought about last in the design of them, and then the canteen which the public use as well, they’ve now taken away of all the table and chairs. So, I don’t know if management have actually thought about where well people are eating their lunch. (Hospital doctor)

This was felt to be particularly acute to those who worked out of hours:

Basically what it’s shown is that people who work shifts and work in hospitals need to have access to rest spaces. And they just don’t exist. There’s something for NHS Scotland, give proper rest spaces to staff and provide free tea and coffee. But its about making them feel a bit more valued. (Hospital doctor)

The discussion of this group of participants also hints at a hidden logic of healthcare professionals having no control over their professional work environment. There was a clear sense that staff wellbeing was not an important consideration when planning healthcare provision.

And there isn’t any. Now we’ve asked for it repeatedly and partly because the buildings are not owned by the board, then it just gets again into that too difficult. There are large endowment funds in lots of hospital that you can’t access for anything at all, but I do think wellbeing spaces might be one of those things that could come under those rules. (Hospital doctor)

Not all areas had such negative experiences associated with space provision, with some organisations appearing to be prompted by COVID-19 to acknowledge the importance of these spaces within the workplace environment. The previous barriers (ie, financial costs and logistics on space) to the implementation of safe spaces were removed.

Because we had a quite well-developed wellbeing group……and she said to me one day about two or three years ago, oh, wouldn’t it be good for us to have a breakout spaces where you could go and do mindfulness during the day. And so, for the last three years we’ve being trying to identify the space and get funding, then all of a sudden, like everything, all the red tape has come away and we’ve managed to get this mindfulness space. Its actually been launched in the last month. (Hospital doctor)

## Discussion

Throughout the UK, the pressure on staff, especially doctors, to deliver services has intensified as the NHS tries to transition beyond the COVID-19 pandemic. This study explores the provision of R&R spaces, since they can support doctors’ wellbeing, perceptions of being valued by the organisation, workforce retention and ultimately patient safety. The pandemic may have passed, but the NHS is still in crisis and the lessons of this study have urgent implications for the present day and beyond.

As demonstrated in this study, II offers a valuable means of unpicking some of the challenges of enacting policy changes within the healthcare setting. One-size-fits-all initiatives, such as R&R spaces, are laudable, but unless local context is taken into account, the enactment of change is loosely coupled with policy. II allowed us to highlight areas of discrepancy between individual and organisation perceptions of the provision of R&R spaces and theorise reasons for this. Numerous disparities were apparent between what was decreed at HB level and how hospital doctors experienced these initiatives. This disparity could also be described as the differences between ‘work as imagined’ and ‘work as done’.^29^ We identified that the suitability of provision for purpose was a key factor in supporting doctors in a meaningful way. However, despite the HBs indicating they used staff communication to support wellbeing, this appears to be ineffective, resulting in wasted opportunities and lack of potential benefit for staff. II allows us to see that the messaging of change was too tightly coupled with policy at an organisational level. A greater understanding of such discrepancies in perception could facilitate learning and best practice for current and future initiatives to support health professional well-being.

No access to R&R spaces has negative implications on doctors’ sense of belonging within their workplace—a sense of not feeling like they fit in anywhere and have nowhere to go. This feeds into the notion that they are not valued, appreciated and supported appropriately by the organisation. The logic being that organisations provide services without any regard for the utility of the provision. However, for junior doctors, having access to these spaces may also facilitate their integration into the medical community and develop a sense of belonging with the profession and strengthen their professional identity as a healthcare professional.^30^ These spaces are not just about wellbeing; they can facilitate an integration process and provide a community for doctors across the career continuum to come together, receive support and share experiences.

In addition, hospital doctors in our study voiced a reasonable expectation for organisations to provide a sanctuary to support them in facing the inevitable challenges of their daily work. Although this study focused on R&R spaces and doctors, the principles could be extrapolated into the consideration of safe social spaces and resultant benefits for all staff. The wider literature has shown that employees who have positive workplace relationships and job enjoyment/satisfaction with the organisation are more likely to be committed to the organisation and less likely to leave.^31 32^ Unintentionally or not, the institutional logic identified was that their employing organisation put the onus of wellbeing firmly at the individual level. This needs to be reversed, and the culture changed so organisations are not only aware of their responsibilities but also provide a channel for hearing what is needed and where.

This theory-informed national study aimed to explore issues central to the wellbeing, productivity and retention of hospital doctors across the career continuum. This large-scale study produced findings of particular relevance given the current pressures on the healthcare system. The interviews with the hospital doctors were completed prior to receiving all of the information from the HBs, since they were surveyed after the COVID-19 pandemic and asked to reflect on past, present and future service provision. The recruitment of the hospital doctors to the study was based on voluntary participation, which has introduced self-selection bias, potentially over-representing individuals with unresolved concerns. Additionally, all data were self-reported, which may have introduced further bias. Future research would benefit from incorporating observational methods or third-party corroboration to strengthen the validity of the findings.

COVID-19 shone a light on the importance of wellbeing for staff,^7 33^ although many of the pressures such as an overwhelming workload were not new or unrecognised.^4 34^ For some, previous barriers (ie, red tape) to the implementation of wellbeing initiatives disappeared almost overnight. However, it has also been shown that these wellbeing initiatives can be removed (and in some cases have been) just as quickly. With the current pressure of healthcare budgets, is there a risk that investment in wellbeing initiatives, such as support for development and maintenance of R&R spaces, will be withdrawn to support frontline patient care?^34 35^ We would argue that this is a false economy as a healthy workforce has been shown to lead to improved patient outcomes, fewer patient errors, higher levels of job satisfaction, commitment to the organisation and workforce retention.^36^ ^37 38^ Aware of the current financial constraints, hospital doctors are not looking for grandiose gestures, but spaces where they can store their personal belongings, a place to hydrate and refuel, escape workplace pressures and share camaraderie with their colleagues, something that would be a basic necessity in organisations outwith healthcare.

## Conclusions

Greater understanding of differences in perception could facilitate learning and best practice for current and future organisational initiatives to support health professional wellbeing.

Organisations must consider how they communicate with the clinicians about non-clinical issues, such as the provision of R&R spaces. This study illustrated that just using traditional global emails or assuming the information is fed through line managers is not reaching doctors, especially trainees. In this era of technology and the use of social media, it may be that these methods may prove more productive in advising what is available and where and allowing an opportunity to feedback when and why it is not working for them. It is imperative our findings are used to both prompt and inform further action in this important area at a local and organisational level, recognising the need for these levels to work in partnership to address this need. Only then can we care for our healthcare professionals as they endeavour to care for others.

## Supplementary material

10.1136/bmjopen-2024-096154 online supplemental file 1

## Data Availability

Data are available upon reasonable request.
