# Supplementary material for: Investigating discrepancies in perceptions regarding the provision of hospital rest and relaxation spaces in Scotland during the COVID-19 pandemic and beyond: a qualitative study
Source: BMJ Open. 2025 Aug 12;15(8):e096154. doi: 10.1136/bmjopen-2024-096154 (PMC12359540; doi:10.1136/bmjopen-2024-096154)
Supplement: online supplemental file 1 [file bmjopen-15-8-s001.docx]

Dear xxxx

We are a research collaboration, between the five Scottish Medical Schools and NHS Education for Scotland, with expertise in wellbeing, intervention development and evaluation, who have been granted funding by the CSO to develop evidence-based interventions to support wellbeing and promote resilience amongst doctors in NHS Scotland.

To date we have collected data on COVID-19-related experiences and wellbeing from 100 doctors located in every region across Scotland across a range of career grades. A prioritisation process involving expert and stakeholder representation alongside the research team has prioritised five evidence-based interventions for development in order to support doctors’ wellbeing and promote resilience.

We are writing to you to explore the possibility of your Board / site becoming a test location for one of the interventions: the provision of rest and relaxation (R&R) spaces for doctors. We are aware that some R&R spaces provided during the emergency phase of COVID-19 have now been removed or are less available. However, based on our evidence from interview and audio diary data collected from doctors across Scotland, we strongly believe that reintroduction of an R&R space within the hospital warrants serious consideration.

As highlighted in the attached evidence document, these spaces are perceived as critical environments that enable doctors to decompress, rest, and recuperate and provide an opportunity for reducing feelings of isolation. Moreover, these spaces provided a safe space to discuss challenges and struggles with peers or colleagues. Importantly, they also enhance perceived feelings of organisational commitment to and investment in staff wellbeing.

The reintroduction of these spaces, from Boards and hospitals where they have been removed, is of particular interest to us since this would enable a ‘before and after’ evaluative study to be conducted. This type of evaluation is important in highlighting any shifts in wellbeing amongst staff and will enable us to help Boards understand how they may best invest in staff wellbeing during this pandemic and beyond

Accordingly, we ask if you would please consider reintroducing an R&R space in the hospital

Thank you for taking the time to read our thoughts and findings associated with the reimplementation of wellbeing spaces in your hospital. From the evidence presented in the proceeding pages, we hope, you will be able to see the significant benefits of reimplementation of these spaces for your staff’s wellbeing. We would really like to explore this idea with you and will be in contact shortly about arranging an appropriate time to speak further about this.

Yours sincerely,

Dr Kim Walker

Principal Investigator (on behalf of the SMERC Project Team)

What our evidence-base says

We outline our evidence-base for the importance of the reintroduction of R&R space structured around three key areas: the importance of supporting wellbeing and resilience; the considerable benefits of R&R spaces for doctors; the significant negative impact of taking away R&R spaces has on doctors.

Need to support wellbeing and promote resilience

Doctors’ wellbeing and resilience is a key issue facing the medical workforce. Doctors in our study confirmed this by highlighting their struggles with their mental wellbeing, the stigma surrounding disclosure and the need for there to be mechanisms in place to help prevent worsening of mental wellbeing (as highlighted in the quote below). Worsening of mental wellbeing may result in both absenteeism and presenteeism of employees having a significant effect on workforce capacity in addition to both staff and patient safety.

‘I felt quite a lot of shame from the fact that I wasn’t well enough to work, I wasn’t able to concentrate on what I was doing, I wasn’t really able to eat or sleep… It’s something I've become quite passionate about, talking about with people and I actually struggle to think of a single doctor who hasn’t opened up about some form of experience they’ve had whether during Covid or beforehand, of their difficulties with their mental health and the expectation that you shouldn’t suffer somehow, that you should somehow be immune to it. I think hopefully that’s kind of falling out of fashion a bit, but there is still a lot of stigma surrounding it...’

Benefits of rest and relaxation spaces to wellbeing

Doctors reported that they felt that R&R spaces were crucial in promoting wellbeing and resilience amongst staff. Provision of such spaces was perceived by doctors as demonstration that the organisation was committed to staff wellbeing, and this investment promoted the feeling within staff that they were valued and appreciated by the organisation. R&R spaces provided an opportunity for staff to escape to relax and rest, have breaks, undertake debriefs and discuss their experiences including challenges and struggles with their peers – all critical to staff wellbeing and resilience. R&R spaces provided a place for staff to reduce feelings of isolation when working alone or during night shifts. Establishing connectedness with others and accessing social support is particularly important in promoting wellbeing. The provision of psychological support from a trained professional was also perceived by doctors to be important and helpful, not only in promoting access to mental health services within the medical workforce, but also in terms of demonstrating organisational commitment to staff wellbeing (as highlighted in the quote below).

‘That’s just there’s a room that’s a place that staff can go if they want to take a break and the one at the main hospital, there’s counselling staff there all the time, well, for most of the hours of the day. That’s available to the acute staff but also to us as well. We have used that, I think most of us have kind of gone up at least once, I've just gone up once, once on a weekend when I was going up to get lunch from the main campus. That’s I think a good sign of the organisation actually wanting to be supportive’

Impact of taking away rest and relaxation spaces

Doctors who had access to R&R spaces during the height of the first wave of the COVID-19 pandemic and who consequently had them removed in their place of work, were disappointed and saddened by this decision by the organisation. The removal of these facilities made these individuals feel the organisations commitment to wellbeing was disingenuous and short lived. The resultant feelings amongst some doctors were that they did not feel prioritised, or appreciated by the organisation (as highlighted in the quote below).

‘Those were nice positive changes to see. And I think it’s a shame to have to suddenly see them rolled back on quite so rapidly. It’s almost a bit like, “Well, that was your small benefit you got from coronavirus, but we didn’t really it mean it for long-term, so we’re taking that away again.” It does seem a little bit disingenuous and then Boards interest in wellbeing it feels like they’ve taken a step back again’

Reintroduction of R&R spaces

The next phase of our study involves evaluating implemented interventions. We ask if you would please consider reintroducing an R& R space within the hospital and allow us to determine the impact of that reintroduction on doctors’ wellbeing. In conducting this research, we can help Boards understand how they may best invest in staff wellbeing during this pandemic and beyond. Understanding this is critical since doctors are facing increased levels of burnout and reduced mental wellbeing as a consequence of pressures and stressors associated with COVID-19.

Large scale burnout poses a significant threat to organisational sustainability and workforce planning since it forces many to reconsider careers or moving to pursue a career elsewhere, perhaps abroad. ^(1)^ Recent surveys of doctors^(2-6)^ demonstrate that these are both very real threats and hence, there is a very desperate need to call to action. Furthermore, reduced wellbeing or burnout, has also been associated with poor patient safety outcomes.^(7)^ Accordingly, prioritisation of doctors’ wellbeing has the potential to also positively impact on patient safety.

**References**

1. Scanlan, G.M., Cleland, J., Walker, K. and Johnston, P., 2018. Does perceived organisational support influence career intentions? The qualitative stories shared by UK early career doctors. *BMJ open*, *8*(6).
2. Lemaire JB & Wallace JE (2017) Burnout among doctors. BMJ 358: j3360.
3. Dyrbye LN, West CP, Satele D, et al. (2014) Burnout among U.S. medical students, residents, and early career physicians relative to the general U.S. population. Academic Medicine 89:443-51.
4. Rogers ME, Creed PA & Searle J (2014) Emotional labour, training stress, burnout, and depressive symptoms in junior doctors. Journal of Vocational Education & Training 66:243-248.
5. Bernburg M, Vitzthum K & Groneberg DA (2016) Physicians' occupational stress, depressive symptoms and work ability in relation to their working environment: a cross-sectional study of differences among medical residents with various specialties working in German hospitals BMJ Open 6:e011369. doi: 10.1136/bmjopen-2016-011369.
6. Lachish S, Goldacre MJ & Lambert T (2016) Associations between institutional support, job enjoyment, and attitudes to work in first-year doctors in the UK: national questionnaire survey. BMC Med Education 16:151. doi: 10.1186/s12909-016-0673-6.
7. Hall LH, Johnson J, Watt I, Tsipa A, O’Connor DB. Healthcare staff wellbeing, burnout, and patient safety: a systematic review. PloS one. 2016 11(7).
