# Supplementary material for: Investigating discrepancies in perceptions regarding the provision of hospital rest and relaxation spaces in Scotland during the COVID-19 pandemic and beyond: a qualitative study
Source: BMJ Open. 2025 Aug 12;15(8):e096154. doi: 10.1136/bmjopen-2024-096154 (PMC12359540; doi:10.1136/bmjopen-2024-096154)
Supplement: Supplementary file 2 [file bmjopen-2024-096154.reviewer_comments.pdf]

PEER REVIEW HISTORY

BMJ Open publishes all reviews undertaken for accepted manuscripts. Reviewers are asked to complete a checklist review form and are provided with free text boxes to elaborate on their assessment. These free text comments are reproduced below.

ARTICLE DETAILS

Title (Provisional)

Investigating discrepancies in perceptions regarding the provision of hospital Rest and Relaxation spaces in Scotland during the COVID-19 pandemic and beyond: A qualitative study

Authors

Walker, Kim; Cunningham, Kathryn B; Ferguson, Julie; Gibson Smith, Kathrine; Scanlan, Gillian Marion; Cecil, Joanne E; Gordon, Lisi; Laidlaw, Anita; Pope, Lindsey Margaret; Johnston, Peter; Aitken, Gill

---

VERSION 1 - REVIEW

**Reviewer**	**1**
**Name**	**aqtam, ibrahim**
**Affiliation**	**Ibn Sina College for Health Sciences, Nursing**
**Date**	**28-Nov-2024**
**COI**	**None**

This is a very well-designed and thoughtfully conducted study that provides a very valuable insight into the disconnect between institutional policies and staff experiences in healthcare settings. Recommendations have the potential to significantly contribute to the development of policies aimed at improving the wellbeing and retention of staff. The proposed suggestions, if addressed, will further strengthen the paper and enhance its impact.

Thank you for contributing this important work to the field!

Review and Proposed Changes

Title and Abstract:

The title certainly reflects the main idea being researched. Nevertheless, it is a bit wordy and might be more effectively phrased with a view to clarity and punch, for example: "Staff Wellbeing Neglected? Investigating Discrepancies in Rest and Relaxation Space Provision during the COVID-19 Pandemic."

The abstract is good in summarizing the research, but it could be smoothed out for easier reading, particularly with respect to methodology.

Introduction

The introduction provides a clear rationale for the study in linking staff wellbeing to organizational outcomes.

It would be helpful to indicate briefly those specific gaps in the literature that this study addresses. For example, emphasize the lack of comprehensive data on how organizations' claims align with the experience of staff.

Methods:

The application of "Inhabited Institutionalism" is highly relevant and should be explained briefly. Please provide a short description of why this theory applies especially to this study.

Sampling for hospital doctors and health boards needs further explication. Was there a criterion for inclusion other than availability?

Results:

The results are quite comprehensive but would have been easier to understand if the subheadings separated the findings related to the health boards from those related to individual doctors.

Discussion

The discussion effectively ties findings to broader implications, such as staff retention and wellbeing. However, it could benefit from a stronger focus on actionable recommendations for organizations.

Emphasize how communication is a recurring theme and give more specific recommendations as to how this gap could be filled, such as making full use of digital tools for real-time updates on the availability of R&R space.

Strengths and Limitations:

This study's national scope and its theoretical framework are strengths but acknowledge that reliance on self-reported data from health boards and doctors might introduce bias.

Suggest how future studies could make use of observational methods or third-party assessments for corroboration.

Conclusion:

The conclusion summarizes the study quite well but could re-emphasize the urgency in addressing identified gaps, particularly for post-pandemic recovery planning.

Reviewer 2

Name Zurynski, Yvonne

Affiliation Macquarie University, Australian Institute of Health Innnovation

Date 23-Dec-2024

COI None

I found this paper very interesting and informative, however, a number of issues need to be addressed and clarified, before the paper can be considered for publication.

The strengths and limitations summary need to be revised to include study limitations. Explain how being supported by a large team is a strength in this context and what exactly were the standardised and validated methods in this qualitative study?

The definition of R&R has been provided in the introduction, however, is this a definition developed by the authors or can you point to an accepted definition in the literature? Where does the terminology of "wellbeing hub, wobble room" etc. come from? Page 5 of 20 line 21

The last sentence of the 1st paragraph on page 6 of 20 line 10-13 doesn't quite follow what was said in that paragraph.

The relationship between the current study and the previous study needs to be clarified throughout - the introduction seems to overlap in parts with the methods. There is some confusion about sources of data and exactly how data previously collected in the Scottish Doctors Wellbeing Study is being analysed for this study.

The dissonance between doctors and HB representatives is described in the introduction - isn't this the main result of this study?

The introduction would benefit from a greater exploration and setting the scene in terms of policy - is the provision of R&R spaces be a routine responsibility of the NHS and HBs and one of the rights of employees? Is there are charter of employees that you can refer to?

The context and setting of this study needs to be clarified, especially the details about HBs and their scope in care delivery - this is quite important for an international readership who may not be intimately familiar with the way NHS Scotland is organised and governed. The questions appear to be about Hospital spaces only, but this hasn't been explained and reference is made to HBs as a whole. Scotland is a large and diverse country with a great variety of urban rural and remote regions - were doctors only from city hospitals recruited?

When you say HBs were contacted - who exactly was contacted in the HB? page 7 of 20, line 8.

The use of the Inhabited Institutionalism concept provides a very helpful theoretical lens, however, the discussion barely mentions this theory - perhaps the link between II and your results could be more explicitly explained?

The description of the analytical approach needs to be clarified. What exactly is meant by "top down" and "bottom up"? what resources were used to guide the analytical methods and approaches? page 8 of 20 line 46. Content analysis tends to suggest a deductive approach —especially the use of categories and subcategories to describe the content is usually focussed on the reoccurrences of concepts or keywords at a more surface-level of analysis than the more in-depth thematic analysis approach which lends itself more easily to an inductive approach. There appears to be very little (if any) evidence of inductive work in the analysis presented in this paper. Please clarify what was done for data analysis.

The micro, meso and macro levels of institutions are mentioned without any definition and this is never mentioned again. What is the purpose of this? Page 9 of 20 line 36

In the methods section Please explain what is happening during "interview 1" and "interview 2" and at what time points these were undertaken and with whom exactly?

Was "interview 2" collected from the same participants as "interview 1" - that is the same cohort was followed longitudinally or are they different participants at "interview 2". Perhaps a diagram showing the study design and data collection flows would be helpful.

Please clarify whether participants received a participant information sheet explaining the study, and whether they signed a consent form.

A description of the doctor demographics is needed - what contexts did they work in? (page 10 of 20 line 8) It's very different working in a busy metropolitan ED compared with a remote country hospital... Were contextual factors such as this taken into account when interpreting data?

Please clarify in the results: Doctors from how many of the 14 HBs were interviewed and how many said they had no access to R&Rs? You need to demonstrate the dissonance a bit more clearly in the results.

What is "just over half" - provide n and % (page 10 of 20 line 8)

Who were the organisational participants from HBs? Who actually provided the information? This needs to be clear, especially with regards to Table 2.

The quote on page 13 of 20 line 20 refers to "... across staff groups" implies staff groups other than doctors, and yet this study is about hospital doctors only - how do you reconcile this?

The points about cafes being used as R&R spaces needs context - were these spaces available to staff even during the pandemic - were there no restrictions and lock-downs to these areas in Scotland?

Consider shortening the quotes on pages 15 - 16.

The discussion could be strengthened by linking more strongly with II and comparing the results with exiting NHS policy (see comments above).

Page 16 of 20 line 34: The concept of Work-as-imagined and Work-as-done may be a helpful concept at this point in the discussion - Jeffrey Braithwaite, Robert L. Wears, Erik Hollnagel, Resilient health care: turning patient safety on its head, International Journal for Quality in Health Care, Volume 27, Issue 5, October 2015, Pages 418–420, https://doi.org/10.1093/intqhc/mzv063

The discussion feels unfinished. What should happen next in terms of research and policy development and implementation? Please add a section on strengths and limitations of this study into the discussion. Please add a paragraph of conclusions.

Style and terminology:

The terminology needs to be made more consistent throughout this manuscript to improve clarity. Examples include: the study is clearly about hospital doctors but sometimes you refer to them as "staff" which has a much broader meaning; dissonance and discordance seem to be used interchangeably but different terms have been used in different sections.

Clarity of expression could also be reviewed and improved e.g. page 13 of 20 line 7

Please review your style - numerals should not be used at the beginning of sentences (e.g. page 10 of 20 line 19); numbers <10 in the text should be written in words etc. e.g. Two rather than 2.

Please ensure that all quotes are appropriately and consistently formatted.

VERSION 1 - AUTHOR RESPONSE

Reviewer 1 comment	Response
1. This is a very well-designed and thoughtfully conducted study that provides a very valuable insight into the disconnect between institutional policies and staff experiences in healthcare settings. Recommendations have the potential to significantly contribute to the development of policies aimed at improving the wellbeing and retention of staff. The proposed suggestions, if addressed, will further strengthen the paper and enhance its impact.	Thank you very much for these kind words.
2. The title certainly reflects the main idea being researched. Nevertheless, it is a bit wordy and might be more effectively phrased with a view to clarity and punch, for example: "Staff Wellbeing Neglected? Investigating Discrepancies in Rest and Relaxation Space Provision during the COVID-19 Pandemic."	Thank you for your suggestion which has been used in the modified title.
3. The abstract is good in summarizing the research, but it could be smoothed out for easier reading, particularly with respect to methodology.	Thank you. The abstract has been amended to make the methodology clearer and hopefully more readable.

4. The introduction provides a clear rationale for the study in linking staff wellbeing to organizational outcomes. It would be helpful to indicate briefly those specific gaps in the literature that this study addresses. For example, emphasize the lack of comprehensive data on how organizations' claims align with the experience of staff.	Thank you for this comment. A scoping review of the evidence and the lack of it has been referenced in the manuscript. The comment relating to this has been added to the introduction to emphasise this point.
5. The application of "Inhabited Institutionalism" is highly relevant and should be explained briefly. Please provide a short description of why this theory applies especially to this study	Further information relating to inhabited institutionalism and how it relates to the study has been added on page 8.
6. Sampling for hospital doctors and health boards needs further explication. Was there a criterion for inclusion other than availability?	The sampling for the doctors has been made more explicit and also linked to reference 5 which details the larger SDWS in a new context section. All the Health Boards were involved which we have stated in the manuscript.
7. The results are quite comprehensive but would have been easier to understand if the subheadings separated the findings related to the health boards from those related to individual doctors.	Thank you for this suggestion. Sub headings have been inserted into the results section
8. The discussion effectively ties findings to broader implications, such as staff retention and wellbeing. However, it could benefit from a stronger focus on actionable recommendations for organizations.	Thank you for this suggestion. Further sentences have been inserted into the discussion to try and emphasise this more.
9. Emphasize how communication is a recurring theme and give more specific recommendations as to how this gap could be filled, such as making full use of digital tools for real-time updates on the availability of R&R space	The conclusions have been strengthened with comments about how communication could be improved in line with the reviewers suggestions.
10. This study's national scope and its theoretical framework are strengths but acknowledge that reliance on self-reported data from health boards and doctors might introduce bias. Suggest how future studies could make use of observational methods or third-party assessments for corroboration	Thank you for highlighting this. Your suggestion has been added into the main text.
11. The conclusion summarizes the study quite well but could re-emphasize the urgency in addressing identified gaps, particularly for post-pandemic recovery planning	An extra line has been added to the conclusions which hopefully addresses this comment.

Reviewer 2 comments	Response
1.I found this paper very interesting and informative, however, a number of issues need to be addressed and clarified, before the paper	Thank you for your kind comment about the paper being very interesting and informative.

can be considered for publication.	
2. The strengths and limitations summary need to be revised to include study limitations. Explain how being supported by a large team is a strength in this context and what exactly were the standardised and validated methods in this qualitative study?	The strengths and limitation have been revised – new limitations have been added. A statement explaining the strength of a large team has also been added. To avoid confusion, the comments about standardised and validated methods has been removed.
3. The definition of R&R has been provided in the introduction, however, is this a definition developed by the authors or can you point to an accepted definition in the literature? Where does the terminology of "wellbeing hub, wobble room" etc. come from? Page 5 of 20 line 21	Thank you for your comments. We have added the following reference to the definition we provided which highlights the key components of such spaces: https://asmepublications.onlinelibrary.wiley.com/doi/epdf/10.1111/medu.14946<br><br>We have also added the following references which highlights other terminologies used for these types of spaces: https://www.england.nhs.uk/supporting-our-nhs-people/support-now/staff-mental-health-and-wellbeing-hubs/<br>https://wellbeinghub.scot/<br>https://www.bmj.com/content/371/bmj.m4461
4. The last sentence of the 1st paragraph on page 6 of 20 line 10-13 doesn't quite follow what was said in that paragraph.	Thank you for pointing this out. The last sentence in this paragraph has been moved further up the paragraph so that is now reads better.
5. The relationship between the current study and the previous study needs to be clarified throughout - the introduction seems to overlap in parts with the methods. There is some confusion about sources of data and exactly how data previously collected in the Scottish Doctors Wellbeing Study is being analysed for this study.	Thank you for your comments. In order to clarify the relationship between this study and the SDWS study, a context section has been added. Some sections of the introduction have been moved to this section. There is also more specific reference to a previous publication which contains information relating to the earlier study.
6. The dissonance between doctors and HB representatives is described	Thank you for this comment. The paper has been edited and information moved from the introduction

in the introduction - isn't this the main result of this study?	
7. The introduction would benefit from a greater exploration and setting the scene in terms of policy - is the provision of R&R spaces be a routine responsibility of the NHS and HBs and one of the rights of employees? Is there are charter of employees that you can refer to?	Thank you for these comments. We have added reference to the BMA Charter as suggested. Furthermore, further references and sentences comments have been added into the introduction. These are linked to item 3 of your comments. Further references have also been added into the discussion.
8. The context and setting of this study needs to be clarified, especially the details about HBs and their scope in care delivery - this is quite important for an international readership who may not be intimately familiar with the way NHS Scotland is organised and governed. The questions appear to be about Hospital spaces only, but this hasn't been explained and reference is made to HBs as a whole. Scotland is a large and diverse country with a great variety of urban rural and remote regions - were doctors only from city hospitals recruited?	Thank you for this comment. A new context section has been added to address this. The doctors came from across all hospitals in Scotland. It is acknowledged these can be different. However, the results indicate that regardless of location, the same result of differences in perception were noted.
9. When you say HBs were contacted - who exactly was contacted in the HB? page 7 of 20, line 8.	The information relating to the organisational participants has been added to the main text in the methods. In order to ensure anonymity, titles have been provided rather than individual names. This links to item 18.
10. The use of the Inhabited Institutionalism concept provides a very helpful theoretical lens, however, the discussion barely mentions this theory - perhaps the link between II and your results could be more explicitly explained?	Thank you for this comment. Further information has now been added to address this in the discussion.

11. The description of the analytical approach needs to be clarified. What exactly is meant by "top down" and "bottom up"? what resources were used to guide the analytical methods and approaches? page 8 of 20 line 46. Content analysis tends to suggest a deductive approach —especially the use of categories and subcategories to describe the content is usually focussed on the reoccurrences of concepts or keywords at a more surface-level of analysis than the more in-depth thematic analysis approach which lends itself more easily to an inductive approach. There appears to be very little (if any) evidence of inductive work in the analysis presented in this paper. Please clarify what was done for data analysis.	Thank you for these comments. In order to clarify the analytical approach, the words “top down and bottom up” have been removed, the text of the manuscript adjust and examples of deductive and indictive codes provided. The resources are references which have now been added into the text.
12. The micro, meso and macro levels of institutions are mentioned without any definition and this is never mentioned again. What is the purpose of this? Page 9 of 20 line 36	Thank you for this comment. The levels of institutions are as described in II and was intended to show the complexities involved in working in an organisation.. However, in order to provide further clarity, we have removed these terms and inserted “senior and middle management” to more accurately reflect the multiple roles that are involved.
13. In the methods section Please explain what is happening during "interview 1" and "interview 2" and at what time points these were undertaken and with whom exactly?<br>Was "interview 2" collected from the same participants as "interview 1" - that is the same cohort was followed longitudinally or are they different participants at "interview 2". Perhaps a	Thank you. In the methods, a new section has been added to provide the context for the study and reports on the interviews. There is also a reference to our published paper (reference 5) which has this information in detail.

diagram showing the study design and data collection flows would be helpful.	
14. Please clarify whether participants received a participant information sheet explaining the study, and whether they signed a consent form.	This information has been included in the paragraph entitled ethics approval and consent to participate. This links to editors comments, item 4.
15. A description of the doctor demographics is needed - what contexts did they work in? (page 10 of 20 line 8) It's very different working in a busy metropolitan ED compared with a remote country hospital… Were contextual factors such as this taken into account when interpreting data?	Thank you for this comment. The demographic data collected as part of the wider study did not include actual locations as this could lead to identification of individuals. Therefore, only the name of the Health Board was recorded. The boards across Scotland include large urban hospitals as well as smaller more rural provision. While it is appreciated that there are differences between the types of hospital, due to the large amount of data from across all boards, it is considered from the analysis that these conclusions can be drawn.
16. Please clarify in the results: Doctors from how many of the 14 HBs were interviewed and how many said they had no access to R&Rs? You need to demonstrate the dissonance a bit more clearly in the results.	Further information has been provided in the text to clarify how many doctors from how many health boards were interviewed. We have reiterated how many stated they had no access to R&R's. This is a qualitative study of the doctors responses, rather than answering direct questions as per the Health Boards. Therefore content analysis was done with no quantitative analysis.
17. What is "just over half" - provide n and % (page 10 of 20 line 8)	In response to your question, we have provided actual numbers.
18. Who were the organisational participants from HBs? Who actually provided the information? This needs to be clear, especially with regards to Table 2.	The information relating to the organisational participants has been added to the main text in the methods. In order to ensure anonymity, titles have been provided rather than individual names. This links to item 9.
19. The quote on page 13 of 20 line 20 refers to "… across staff groups" implies staff groups other than doctors, and yet this study is about hospital doctors only - how do you reconcile this?	The study was in relation to doctors. However, many of the Health Boards in response to the questions we posed, the Chief Executives and NHS Directors because they oversee the whole Board and staff provided information on R&R spaces in the hospital environment for all staff groups and not soley doctors. Therefore, the quote has been reported verbatim as it highlights a point. It does cover all staff but is relevant to doctors as well.

20. The points about cafes being used as R&R spaces needs context - were these spaces available to staff even during the pandemic - were there no restrictions and lock-downs to these areas in Scotland?	We have clarified this point in our results to read 'However, doctors mostly considered a R&R space to be 'backstage' locations", somewhere away where patients did not have access to.' This point was unrelated to COVID restrictions but was more related to the fact that these definitions of spaces were related to locations that patients were not able to access at any point in time. (https://asmepublications.onlinelibrary.wiley.com/doi/epdf/10.1111/medu.14946
21. Consider shortening the quotes on pages 15 - 16.	Thank you for this suggestion. The quotes have been shortened.
22. The discussion could be strengthened by linking more strongly with II and comparing the results with exiting NHS policy (see comments above).	Thank you. The discussion has been amended and further text added to address this comment and the ones above.
23. Page 16 of 20 line 34: The concept of Work-as-imagined and Work-as-done may be a helpful concept at this point in the discussion - Jeffrey Braithwaite, Robert L. Wears, Erik Hollnagel, Resilient health care: turning patient safety on its head, International Journal for Quality in Health Care, Volume 27, Issue 5, October 2015, Pages 418–420, https://doi.org/10.1093/intqhc/mzv063	Thank you, this concept is relevant to our discussion and we have added the following sentence to highlight this: 'This disparity could also be described as the difference between 'work as imagined' and 'work as done' (https://doi.org/10.1093/intqhc/mzv063
24. The discussion feels unfinished. What should happen next in terms of research and policy development and implementation? Please add a section on strengths and limitations of this study into the discussion. Please add a paragraph of conclusions.	The strengths and limitations to this study have been reported under the relevant section of the paper but have additional points.<br><br>Thank you for your suggestion about the discussion has been amended and additional text added.<br>A conclusion paragraph has been added as requested.
25. The terminology needs to be made more consistent throughout this manuscript to improve clarity. Examples include: the study is clearly about hospital doctors but	Thank you for these comments.<br><br>The terminology has been changed to be more consistent to doctors especially in the results and discussion. Where the findings could be considered to broadened out to include all staff, this has been clearly stated.

sometimes you refer to them as "staff" which has a much broader meaning; dissonance and discordance seem to be used interchangeably but different terms have been used in different sections.	The responses from the FoI requests to the Boards contained information regarding all staff rather than just doctors which is why this has been reported.<br><br>The term discordance has been changed to dissonance to ensure continuity of terms throughout the document.
26. Clarity of expression could also be reviewed and improved e.g. page 13 of 20 line 7	The manuscript has been reviewed and improved to increase the clarity of expression
27. Please review your style - numerals should not be used at the beginning of sentences (e.g. page 10 of 20 line 19); numbers <10 in the text should be written in words etc. e.g. Two rather than 2.	The manuscript has been updated to incorporate this request.
28. Please ensure that all quotes are appropriately and consistently formatted.	The quotes have been checked to ensure they are appropriately and consistently formatted.

VERSION 2 - REVIEW

**Reviewer**	**1**
**Name**	**aqtam, ibrahim**
**Affiliation**	**Ibn Sina College for Health Sciences, Nursing**
**Date**	**19-Feb-2025**
**COI**	

Thank you for the opportunity to re-visit this research paper. The paper is useful and timely analysis of divergence in opinions on availability of Rest and Relaxation (R&R) facilities to hospital doctors and NHS Scotland Regional Health Boards. The paper is well structured with good aims and good theoretical grounding. There are, however, some aspects which can be revised in order to enhance clarity, coherence, and academic integrity.

 The abstract is too long perhaps. Attempt to summarize key findings in a more formal manner.

□ The introduction is a good background but short on focus slightly. Some of the discussion about prior research concerning hospital employee well-being can be reduced.

□ The qualitative approach is fine, but more information on selection of participants and potential biases (e.g., voluntary participation tending to result in self-selection bias) must be added.

□ Explain why a hybrid coding approach was employed and how it supported the building of the thematic analysis.

□ Table 2 is informative but would be easier to show with answers arranged in an identical way.

□ The infographic referred to in the methods must be more fully described or inserted as a figure.

□ The argument can be broadened to cover broader implications beyond Scotland, e.g., comparison with other health systems or global perspectives on staff well-being.

□ Recommendations for policy implementation must be more specific. Instead of "improved communication," for example, recommend specific actions like digital platforms, signage, or R&R coordinators.

□ Anticipate probable counterarguments, i.e., space and budget constraints, and recommend substitute realistic solutions.

□ Some of the paragraphs are too lengthy and should be split for easy reading.

□ Avoid repetition, particularly in discussion and findings.

□ Employ denser wording to force the effect of words.

□ The paper has made use of some words synonymously, i.e., "Rest and Relaxation spaces," "wobble rooms," "staff areas," and "breakout spaces." It would be more concise if the same terms are used throughout the paper.

□ Be more specific near the outset of the paper in defining what an R&R space is and how it is different from other spaces for employees.

□ The application of Inhabited Institutionalism (II) is useful but should be more clearly embedded in the analysis. Specify more clearly how II is applied to inform the analysis of the data.

□ Some references have incomplete information (e.g., missing volume or issue numbers).

VERSION 2 - AUTHOR RESPONSE

Comments – Reviewer 1	Response to comment	Page and line number

1.The abstract is too long perhaps. Attempt to summarize key findings in a more formal manner	Thank you. The abstract has been amended. A reference to the use of II has now been added to the abstract to advise it was integral to the work.	Page 4
2.The introduction is a good background but short on focus slightly. Some of the discussion about prior research concerning hospital employee well-being can be reduced	Thank you for this. We feel it is important to put the study into context for R&R spaces and the benefits of these for wellbeing. However, we have removed a paragraph which hopefully means the introduction is now more focused.	Page 7, lines 45-52
3. The qualitative approach is fine, but more information on selection of participants and potential biases (e.g., voluntary participation tending to result in self-selection bias) must be added.	Thank you for these comments. The reason for the context paragraph in the methods is to reference the paper which outlines in detail how the participants were selected. Reference 5.<br><br>The section entitled "Hospital doctors" also details how the sub set of participants was selected, however we have added a further sentence to clarify even further.<br><br>The potential bias from voluntary participation is stated in the strengths and limitations section.	Page 7, lines 20-22.<br><br>Page 7, lines 36 – 45.<br><br>Page 5, lines 12 - 18
4. Explain why a hybrid coding approach was employed and how it supported the building of the thematic analysis	Thanks for this comment. We think perhaps the reviewer is referring to content analysis. We have therefore amended the text to explain more clearly what we have we did in the analysis.<br><br>This paragraph has now been amended to reflect this comment.	Page 10, lines 14 - 18
5. Table 2 is informative but would be easier to show with answers arranged in an identical way.	Thank you for this comment but we are unclear what the reviewer requires. The answers in the table are the information that was received from the Health Boards which was free text.	Page 12 lines 3 -60<br>Page 13, lines 3 - 42
6. The infographic referred to in the methods must be	Thank you for this comment. The infographic referred to in the Methods, is indicated as Figure 1.	Page 7 line 50.<br>Page 21 lines 33 and 37

more fully described or inserted as a figure.	However, this has now been requested to be removed. Editors comments -number 3	Page 22
7. The argument can be broadened to cover broader implications beyond Scotland, e.g., comparison with other health systems or global perspectives on staff well-being.	Thank you for this. However, since this is a qualitative study, generalisations have to be undertaken with caution. We have attempted to do this in the text of the discussion.	Page 18, lines 45 - 60
8. Anticipate probable counterarguments, i.e., space and budget constraints, and recommend substitute realistic solutions	Thank you. We have highlighted this in the discussion about how lack of prioritisation in R&R spaces and concentration on frontline care is a false economy. Ref. 37,38. We have added in extra text to emphasise this point.	Page 18, lines 44 - 60
9. Some of the paragraphs are too lengthy and should be split for easy reading	Thank you. This has been done in various places.	Page 7 line 16<br>Page 10, line 22 and 44<br>Page 15 line 34 and 50<br>Page 19, line 44
10. Avoid repetition, particularly in discussion and findings.	Thank you. We are keen to keep the detail but have removed some sentences.	Page 17, lines 11 – 27.<br>Page 17, line 59<br>Page 18, lines 3 - 5
11. The paper has made use of some words synonymously, i.e., "Rest and Relaxation spaces," "wobble rooms," "staff areas," and "breakout spaces." It would be more concise if the same terms are used throughout the paper.	Thank you for this comment. We use the term “rest and relaxation spaces” (R&R spaces) consistently throughout the document – abstract, aim, methods, results and discussion.<br><br>We used the words “wobble room” and “relaxation spaces” to explain how the language can be used interchangeably by others and provided references to support this. However, we only used R&R spaces as our terminology.	Page 6, line60<br><br>Page 16.<br><br>Page 13

	The word “breakout space” has only been used in a quotation and therefore we could not change this.<br><br>Similarly “staff area” has only been reported in a quotation and therefore we could not change this.	
12. Be more specific near the outset of the paper in defining what an R&R space is and how it is different from other spaces for employees	Thank you for this comment. We have defined an R&R space in our introduction –<br><br>“*Throughout this paper we use the term R&R space to represent a physical space provided free of charge by the employing institution, offering a physically and emotionally safe environment for hospital staff to freely express themselves without fear of prejudice or negative judgement. In essence, a shared social space or physical space away from patients, their relatives and the public*”<br><br>Our research reported on doctors since they were the participants in our study. Other employees, ie hospital staff could also use these R&R spaces which we have stated in the paragraph above.	Page 6 lines 50 - 60
13. The application of Inhabited Institutionalism (II) is useful but should be more clearly embedded in the analysis. Specify more clearly how II is applied to inform the analysis of the data.	Thank you. The use of the II lens is now listed in the abstract.<br><br>Further detail has been detailed in the methods.	Page 4, lines15 – 20.<br><br>Page 9, lines 22 - 25
14. Some references have incomplete information (e.g., missing volume or issue numbers).	Thank you. The references have been checked again, so that they are a direct link to the publication.	Page 21, line 24 – Page 23, line 30
